# The effect of recovery status and life conditions on the quality of life in colorectal cancer patients with stoma: a path analysis

**DOI:** 10.3389/fpsyg.2025.1693626

**Published:** 2025-12-16

**Authors:** Hande Cengiz Açil, Ayşe Çelik Yilmaz, Dilek Aygin

**Affiliations:** Department of Surgical Nursing, Faculty of Health Sciences, Sakarya University, Sakarya, Türkiye

**Keywords:** colorectal cancer, quality of life, postoperative recovery, stoma, postoperative care

## Abstract

**Purpose:**

This study aimed to examine the relationship between postoperative recovery and quality of life in colorectal cancer patients with a stoma using path analysis.

**Methods:**

The study included 70 colorectal cancer patients who underwent stoma surgery in the General Surgery Department of a university hospital. Of the participants, 58.6% were male, 41.4% female, with a mean age of 62.3 ± 10.8 years. Quality of life was the dependent variable, while psychological activities, physical activities, work/social life, stoma function, bowel symptoms, and desire symptoms were independent variables. Data were collected using standardized scales, and path analysis was applied to assess direct and indirect effects.

**Results:**

Work/social life significantly negatively affected psychological (*β* = −0.41, *p* < 0.01) and physical activities (*β* = −0.36, *p* < 0.05). Both work/social life (*β* = −0.33, *p* < 0.05) and stoma function (*β* = −0.40, *p* < 0.01) negatively influenced desire symptoms. Additionally, stoma function negatively impacted bowel symptoms (*β* = −0.37, *p* < 0.05). The model demonstrated good fit (χ^2^/df = 2.10, RMSEA = 0.07, CFI = 0.94).

**Discussion:**

The findings highlight that stoma-related functional difficulties and disruptions in work/social life may adversely affect psychological and physical recovery, ultimately reducing quality of life. These results support prior evidence suggesting that adaptation to stoma is multifactorial, involving both physical management and psychosocial adjustment. A holistic care approach that addresses these multidimensional challenges is essential.

**Conclusion:**

Tailored education, psychosocial support, and individualized postoperative care can improve adaptation, recovery, and quality of life in stoma patients.

## Introduction

Colorectal cancer is one of the most common malignancies worldwide and often requires surgical intervention as part of the treatment process. In advanced cases, surgical creation of a stoma becomes necessary to divert intestinal contents due to tumor obstruction, perforation, or as part of oncological management. A stoma is a surgically created opening on the abdominal wall that allows the passage of fecal matter, while the procedure itself is termed an ostomy (Şahin and Kumcağız, 2021). Stomas may be temporary or permanent, depending on the disease and treatment plan, and are commonly constructed for patients with colorectal cancer, inflammatory bowel disease, or congenital and traumatic abnormalities (Alptekin and Akyüz, 2024; Ketterer et al., 2021).

The presence of a stoma has profound effects on patients’ physical, psychological, social, and emotional well-being. Physically, patients may experience complications such as fecal leakage, peristomal skin irritation, stoma retraction, constipation, diarrhea, fatigue, and pain (Ketterer et al., 2021; Yan et al., 2022). Psychologically, stoma patients often face anxiety, depression, decreased self-esteem, and body image disturbances (Benfante et al., 2025; Liao and Qin, 2014; Zewude et al., 2021). Socially, altered body appearance, odor, and the fear of stool leakage may lead to social withdrawal and isolation, further reducing quality of life (Zewude et al., 2021; Alenezi et al., 2021). Collectively, these challenges highlight that living with a stoma is not only a medical adjustment but also a psychosocial and lifestyle challenge.

Colorectal cancer treatment itself can additionally impact quality of life. Surgery, chemotherapy, and radiotherapy often cause physical symptoms (e.g., bowel dysfunction, fatigue) and psychological distress (e.g., anxiety, depression) that may interact with stoma-related challenges (Stiegelis et al., 2004; Hernández Blázquez and Cruzado, 2016). Long-term survivors may continue to experience impairments in social and physical functioning, emotional health, and overall quality of life (Jansen et al., 2010; Pachler and Wille-Jørgensen, 2012; Sritan, 2023). Demographic and socioeconomic factors further influence the adaptation to living with a stoma (Kristensen et al., 2019).

Despite increasing research on stoma-related quality of life, there is a paucity of studies exploring the interactions between postoperative recovery and quality of life using advanced statistical approaches such as path analysis. Path analysis can provide insights into the direct and indirect relationships among physical, psychological, and social recovery factors, helping to identify key determinants of adaptation and well-being.

Therefore, the aim of this study is to investigate the relationship and interaction between postoperative recovery and quality of life in colorectal cancer patients with a stoma using path analysis. We hypothesize that physical, psychological, and social recovery variables will have significant direct and indirect effects on overall quality of life, and that stoma-related functional and psychosocial challenges will mediate these relationships.

## Materials and methods

Before starting the study, all participants provided written informed consent, and the study was approved by the Institutional Ethics Committee of [Sakarya University Clinical Research Ethics] (17.03.2022/80).

### Participants

Seventy patients with stoma following colorectal cancer surgery were recruited from the General Surgery Department clinic between 2022 and 2023. Participants included both colon (*n* = 46, 65.7%) and rectal (*n* = 24, 34.3%) cancer patients.

#### Inclusion criteria

- Age ≥ 18 years
- History of colorectal cancer with stoma
- Ability to provide informed consent and complete questionnaires

#### Exclusion criteria

- Severe cognitive impairment or psychiatric disorders that would interfere with questionnaire completion
- Concurrent severe comorbidities that could confound quality-of-life assessment

#### Sampling method

Participants were selected through convenience sampling, whereby patients who met the inclusion criteria were invited to participate.

### Data collection tools

Demographic information: Sociodemographic and clinical data, including age, sex, educational level, type of stoma, were collected via a structured questionnaire.

#### Postoperative Recovery Index (PoRI)

The Postoperative Recovery Index was developed by Butler et al. (2012) and its Turkish validity and reliability was performed by Cengiz and Aygin (2019) and consists of 25 items. The index has 5 sub-dimensions: psychological symptoms, physical activities, general symptoms, bowel symptoms, and appetite symptoms. When calculating the total scale score, all 25 items are summed and the arithmetic mean is taken. In the sub-dimension scores, the sub-dimension score is determined by summing the scores of the sub-dimension items and taking their arithmetic averages. It is determined that the higher the scores obtained from the scale, the lower the recovery, and the lower the scores, the higher the recovery. Assessed using PoRI, measuring physical, psychological, and social recovery domains. To calculate the total score, all items are summed and the arithmetic mean is calculated. Higher scores on the index reflect greater difficulty in postoperative recovery, while lower scores indicate easier postoperative recovery. Original scale reliability: Cronbach’s *α* = 0.96; reliability in the present sample: Cronbach’s α = 0.91 (Butler et al., 2012; Cengiz and Aygin, 2019).

#### Stoma Quality of Life Scale (QoLSS)

The Turkish validity and reliability study of the scale developed by Baxter et al. (2006) was conducted by Karadağ et al. (2011). The scale is a 19-item assessment tool developed to determine the quality of life of individuals with stoma. The first two items of the scale (first part) evaluate the individual’s satisfaction with his/her life on an immediate and monthly basis. The first question shows the current state of satisfaction and may be more effective in evaluating instant emotional changes. The second question reflects satisfaction with the previous month. In the second part of the scale, 17 items are grouped into three sub-dimensions. These dimensions are work/social life (6 items), sexuality/body image (5 items) and stoma function (6 items). The scale is calculated as follows. Each sub-dimension is evaluated out of 100 points (0 = indicates poor quality of life, 100 = indicates good quality of life). A high score on the scale indicates an increased quality of life. Original scale reliability: Cronbach’s *α* = 0.93; reliability in the present sample: Cronbach’s α = 0.71(Baxter et al., 2006; Karadağ et al., 2011).

### Statistical analyses and path analysis

Data were analyzed using IBM SPSS Statistics 23 and IBM SPSS AMOS 23. For categorical variables, frequency distributions (*n*, %) were reported, and for continuous variables, descriptive statistics (mean ± standard deviation) were calculated. Scale reliability was assessed using Cronbach’s alpha coefficients, and relationships between scales were examined with Pearson correlation analysis.

**Figure 1:**
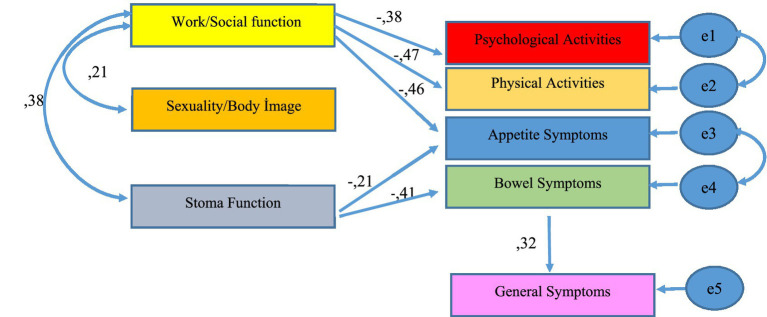
Path model.

To test the proposed model, path analysis was conducted using AMOS 23, with the results reported as standardized regression coefficients. Initially, a path model including eight factor dimensions as observed indicator variables was specified (Figure 1). Since latent variables are not directly metric, either a factor loading of 1 was assigned to one of the paths from each latent variable to its corresponding observed variable, or the variance of the latent variable was fixed at 1, following standard procedures.

Parameters were estimated using the maximum likelihood method, which is commonly employed in structural equation modeling and provides robust estimates even when the data deviate from normality. Estimated parameters included regression coefficients from latent variables to observed variables, variances of latent variables, and errors of observed variables.

To improve model fit, modification indices were examined, and covariances were added between the error terms of the psychological and physical symptoms dimensions, as well as between the cravings and bowel symptoms dimensions, which showed the highest modification indices. Model fit was evaluated using χ^2^/df, RMSEA, CFI, and TLI indices, with statistical significance set at *p* < 0.05.

A post-hoc power analysis was conducted using G*Power 3.1.9.7 to evaluate the statistical power of the study based on the obtained sample size and effect size. For the relationship between the postoperative recovery index and the stoma-related quality of life scale, with an effect size of 0.407 and a significance level of *α* = 0.05, the achieved power was 0.984, indicating that the study was sufficiently powered to detect medium-to-large effects.

Participants were recruited via convenience sampling, meaning that patients who met the inclusion criteria were invited to participate. Overall, these analyses ensured that the statistical methods were rigorously aligned with the study aims and hypotheses, and that the results are statistically robust and replicable.

## Results

The mean age of the participants was 63.96 ± 12.81 years, the mean BMI was 25.56 ± 5.01, 70.0% were male, 61.4% were primary school graduates, 87.1% were married, 31.4% were retired, 65.7% had income equivalent to expenses, 78.6% were non-smokers and 42.9% had temporary colostomy. Participants included both colon (*n* = 46, 65.7%) and rectal (*n* = 24, 34.3%) cancer patients (Table 1).

**Table 1:** Demographic information.

Variable		*n*	%
Age	Mean ± ss (min-max)	63.96 ± 12.81 (24–93)	
BMI	Mean ± ss (min-max)	25.56 ± 5.01 (16.8–50.78)	
Gender	Female	21	30.0
	Male	49	70.0
Postoperative day	Mean ± ss (min-max)	2.07 ± 1.28 (1–9)	
Diagnosis	Colon Cancer	46	65.7
	Rectal Cancer	24	34.3
Marital status	Single	9	12.9
	Married	61	87.1
Education status	Illiterate	9	12.9
	Primary education	43	61.4
	University	5	7.1
	Postgraduate	2	2.9
	High School	11	15.7
Occupation	Not working	10	14.3
	Housewife	15	21.4
	Worker	13	18.6
	Officer	1	1.4
	Retired	22	31.4
	Self-employment	7	10.0
	Other	2	2.9
Income	Income less than expenditure	21	30.0
	Income balances expenditure	46	65.7
	Income more than expenditure	3	4.3
Cigarette smoking	Yes	15	21.4
	No	55	78.6
Alcohol use	No	70	100.0
Stoma type	Permanent colostomy	18	25.7
	Permanent ileostomy	2	2.9
	Temporary colostomy	30	42.9
	Temporary ileostomy	16	22.9
	Permanent colostomy and permanent ileostomy	1	1.4
	Temporary colostomy and temporary ileostomy	3	4.3

The overall mean stoma quality of life score was moderate (55.04 ± 13.47), suggesting that participants experienced a moderate level of stoma-related challenges. The average postoperative recovery index score (2.08 ± 0.70) indicates a medium level of recovery difficulties. Among the subdimensions, the highest mean score was seen in symptoms of general (2.46). Reliability analyses showed acceptable to high internal consistency, with Cronbach’s alpha values exceeding the recommended threshold of 0.70, supporting the reliability of the scales used in this study (Table 2).

**Table 2:** Descriptive statistics for the scale and sub-dimensions.

Scales	ort	ss	Min	Maks
Stoma scale	55.04	13.47	20.56	81.11
Work/social life	45.48	20.70	0.00	87.50
Sexuality/body image	53.64	15.81	20.00	85.00
Stoma function	66.01	21.03	0.00	100.00
Postoperative recovery index	2.08	0.70	1.04	4.24
Psychological symptoms	1.91	0.77	1.00	4.50
Physical activities	2.05	1.17	1.00	5.00
Appetite symptoms	1.86	0.98	1.00	4.75
Bowel symptoms	2.13	0.94	1.00	4.00
General symptoms	2.46	1.24	1.00	5.00

The correlation analysis demonstrated significant negative relationships between the postoperative recovery index and several quality-of-life components. Notably, the stoma scale showed a strong negative correlation with postoperative recovery (r = −0.447, *p* < 0.001), indicating that greater recovery difficulties are associated with poorer quality of life. Work/social life and stoma function were also significantly associated with bowel and general symptoms, highlighting that both social functioning and stoma-related complications play a crucial role in shaping postoperative adaptation (Table 3).

**Table 3:** Examination of the relationship between the scales.

Scales		PoRI	Psychological symptoms	Physical activities	Appetite symptoms	Bowel symptoms	General Symptoms
Stoma scale	*r*	−0.477	−0.179	−0.272	−0.153	−0.367	−0.592
	*p*	0.000*	0.138	0.023*	0.207	0.002*	0.000*
Work/social life	*r*	−0.542	−0.381	−0.475	−0.111	−0.161	−0.542
	*p*	0.000*	0.001*	0.000*	0.360	0.184	0.000*
Sexuality/body image	*r*	−0.177	−0.153	−0.027	−0.025	−0.182	−0.287
	*p*	0.143	0.207	0.822	0.836	0.132	0.016*
Stoma function	*r*	−0.250	0.146	−0.035	−0.165	−0.411	−0.390
	*p*	0.037*	0.227	0.771	0.171	0.000*	0.001*

r: Pearson korelasyon katsayısı, *p < 0.05. Bold values indicates *p < 0.05.

In the last stage, the fit indices for the path model were analyzed. When the findings obtained are examined, it is seen that the path model generally provides a good fit. According to the path model, work/social life measurement has a negative effect on psychological symptoms (*β*: −0.38, *p* < 0.001), work/social life measurement has a negative effect on physical symptoms (*β*: −0.47, *p* < 0.001), work/social life measurement has a negative effect on appetite symptoms (*β*: −0.46, *p* < 0.001), the stoma function measure had a negative effect on the appetite symptoms measure (*β*: −0.21, *p* < 0.045), the stoma function measure had a negative effect on the bowel symptoms measure (*β*: −0.41, *p* < 0.001), and the bowel symptoms measure had a positive effect on the general symptoms measure (*β*: 0.32, *p* < 0.005).

## Discussion

The creation of a stoma significantly impacts patients’ physical, psychological, and social well-being, bringing challenges such as changes in body image, lifestyle limitations, fear of odor or gas leakage, social isolation, reduced self-esteem, sexual dysfunction, and work-related interruptions (Duluklu and Çelik, 2019). Ensuring and improving quality of life (QoL) is therefore a critical goal for patients, caregivers, and healthcare professionals (Berti-Hearn and Elliott, 2019). This study investigated the relationship between quality of life and postoperative recovery in individuals living with a stoma.

In line with previous literature, colorectal cancer survivors may experience persistent physical, psychological, and social challenges despite an overall acceptable quality of life. These difficulties affect daily functioning, emotional adjustment, and long-term health outcomes (Benfante et al., 2025; Jansen et al., 2010; Tesio et al., 2024). Studies have emphasized that QoL in ostomy patients encompasses physical, psychological, social, and spiritual dimensions (Alenezi et al., 2021; Sritan, 2023). In our study, the mean Post-Stoma Quality of Life score was moderate (55.04), consistent with the expectation that individuals in the early postoperative period face significant adjustment challenges. The mean Postoperative Recovery Index score was 2.08, indicating a moderate recovery level. The negative correlation found between postoperative recovery and QoL supports the notion that better recovery is associated with improved stoma-related QoL.

Path analysis was used to explore direct and indirect relationships between psychological, physical, and social functioning. One advantage of path analysis is its ability to evaluate both direct and indirect effects among multiple variables, offering a comprehensive understanding of complex relationships (Lleras, 2005). Our model revealed that impaired work and social life had significant negative effects on psychological symptoms, physical symptoms, and desire-related symptoms. These results indicate that social and occupational limitations may exacerbate psychological distress and physical symptom burden. Stoma-related challenges such as leakage, odor, anxiety, fatigue, and dissatisfaction with appearance can lead to social withdrawal, low self-esteem, and reduced quality of life during the recovery process (Vonk-Klaassen et al., 2016; Boraii, 2017).

Sexual health is also substantially affected by stoma formation. Previous studies have shown that both men and women experience reduced sexual satisfaction, body image concerns, and relationship strain after ostomy formation (Thyø et al., 2019; Alkaya, 2019; Zhu et al., 2017). Consistent with these findings, our results indicated that improved stoma function was positively associated with better work/social life and sexuality scores, suggesting that better control of stoma function contributes to more successful adaptation in personal, social, and intimate areas of life (Ketterer et al., 2021; Davis et al., 2020).

Bowel symptoms such as diarrhea, constipation, gas, and urgency are common in stoma patients and significantly affect daily life and social participation (Altschuler et al., 2018; Miniotti et al., 2022; Reinwalds et al., 2018). In our study, stoma function was negatively associated with bowel symptoms, highlighting that better stoma management reduces bowel-related complications. However, long-term survivors may adjust their lives to these symptoms by modifying daily routines, restricting social interactions, or planning around toilet accessibility (Rutherford et al., 2020).

Overall, the findings emphasize the multidimensional impact of stoma formation on recovery and quality of life, highlighting the importance of comprehensive postoperative care focusing not only on physical healing but also on psychological support, social adaptation, and sexuality counseling. Effective stoma function, ongoing education, psychosocial support, and rehabilitation programs may significantly improve long-term adjustment and quality of life for individuals with a stoma.

## Conclusion

Quality of life in patients with stomas is informed by the personal experiences of important patients. The tools currently available to assess QoL describe what the patient is experiencing and provide insights to the healthcare team regarding QoL outcomes. According to the path model, there is a negative effect of the work/social life measure on the measurement of psychological activities and physical activities, a negative effect of the work/social life measure and the stoma function measure on the measurement of desire-desire symptoms, and a negative effect of the stoma function measure on the measurement of bowel symptoms. As a result, the study revealed that the quality of life of patients with postoperative stoma was at an intermediate level, the stoma function decreased the symptoms of desire and bowel symptoms, and the improvement in psychological and physical activities and desire symptoms had positive effects on work/social life. According to the results of the study, it is recommended to provide training to support patients’ adaptation to the stoma, to follow up the patients after discharge, to identify the problems of the patients and to provide relevant support.

### Relevance for clinical practice

Based on the findings of this study, it is important to understand to what extent the postoperative recovery of patients with stomas affects their quality of life and how information, training and support can be provided to patients in this regard.

## Limitations

This study has several limitations. First, its cross-sectional design prevents the establishment of causal relationships and limits the ability to observe changes over time. Second, the relatively small and culturally homogeneous sample restricts the generalizability of the findings. Additionally, several potentially influential clinical (e.g., tumor stage, adjuvant therapy) and psychological (e.g., coping strategies, personality traits) factors were not included. Finally, all data were collected through self-report questionnaires, which may introduce response bias. Future research should use longitudinal and multicenter designs, including broader clinical and psychosocial variables, and incorporate objective or clinician-rated measures.

## Data Availability

The raw data supporting the conclusions of this article will be made available by the authors, without undue reservation.
